# Oxidative Stress in Neurodegeneration

**DOI:** 10.1155/2011/572634

**Published:** 2011-09-21

**Authors:** Varsha Shukla, Santosh K. Mishra, Harish C. Pant

**Affiliations:** ^1^Laboratory of Neurochemistry, National Institute of Neurological Disorders and Stroke, National Institutes of Health, Bethesda, MD 20892, USA; ^2^Molecular Genetics Unit, Laboratory of Sensory Biology, NIDCR, NIH, Bethesda, MD 20892, USA

## Abstract

It has been demonstrated that oxidative stress has a ubiquitous role in neurodegenerative diseases. Major source of oxidative stress due to reactive oxygen species (ROS) is related to mitochondria as an endogenous source. Although there is ample evidence from tissues of patients with neurodegenerative disorders of morphological, biochemical, and molecular abnormalities in mitochondria, it is still not very clear whether the oxidative stress itself contributes to the onset of neurodegeneration or it is part of the neurodegenerative process as secondary manifestation. This paper begins with an overview of how oxidative stress occurs, discussing various oxidants and antioxidants, and role of oxidative stress in diseases in general. It highlights the role of oxidative stress in neurodegenerative diseases like Alzheimer's, Parkinson's, and Huntington's diseases and amyotrophic lateral sclerosis. The last part of the paper describes the role of oxidative stress causing deregulation of cyclin-dependent kinase 5 (Cdk5) hyperactivity associated with neurodegeneration.

## 1. Oxidative Stress: Oxidants and Antioxidants

Oxidative stress is caused by the imbalance in the production of ROS and the biological system's inability to detoxify those species and repair the resulting damage. The effects of oxidative stress depend upon its impact on the cellular system, whether the system is able to handle well and regain its original state or not. However, high levels of oxidative stress can cause necrosis, ATP depletion, and prevention of controlled apoptotic death [1]. In normal conditions most of the cells are maintained in a reducing environment preserved by enzymes. Any imbalance in the normal redox state leads to toxicity via production of free radicals and peroxides (constituting reactive oxygen and or nitrogen species) damaging proteins, lipids, and DNA of the cell. 

ROS are of two types: less reactive and aggressive. Most of these less reactive species are produced at a low level by normal aerobic metabolism, and the damage they cause to cells is constantly repaired. However, in some instances even the less reactive species like superoxide are readily converted by oxidoreduction reactions with transition metals or other redox cycling compounds into more aggressive radical species that cause extensive cellular damage which includes damage inflicted on DNA [2]. Like in mammalian cells as a consequence of aerobic respiration within the mitochondria, superoxide is generated and sequentially reduced to hydrogen peroxide and hydroxyl radicals which confer a long-term effect on DNA by causing severe damage which ultimately leads to mutations [3]. 

ROS plays an important role in cell signaling, a process termed redox signaling. Thus, to maintain proper cellular homeostasis, a balance must be struck between reactive oxygen production and consumption. Excessive ROS or free radicals need to be either quenched by converting them into metabolically nondestructive molecules or be scavenged/neutralized right after their formation. This protective mechanism is called the antioxidant defense system preventing free radical mediated damage of cells leading to various diseases and aging [4].

The most common naturally occurring anti-oxidants are Vitamin A (retinol), C (ascorbic acid), and E (tocopherol), besides polyphenol antioxidants like flavonoids. Some enzymes also play an important role in combating the oxidative stress and one of the best studied is superoxide dismutase (SOD). Catalase, glutathione peroxidase, aldehyde dehydrogenases, and sulfiredoxin all belong to the class of enzymatic antioxidants [5, 6]. The use of antioxidants to prevent diseases is highly controversial as there are some studies that also suggest a secondary side affect of use of these antioxidants.

## 2. Oxidative Stress and Diseases

As previously mentioned the imbalance between the reactive oxygen/nitrogen species formed and quenched leads to the cell/tissue damage ultimately becoming the cause of many diseases. In humans, oxidative stress is associated with diseases in different ways. Oxidative stress could be generated as a secondary effect of a preexisting diseased condition or could be the central cause of the disease itself. 

Besides being implicated in aging by the accumulation of the ROS and mutations in the mitochondrial DNA (mtDNA), oxidative stress is associated with various pathological conditions. It plays a central role in chronic lung disease (CLD) which is usually found in the preterm infants subjected to high oxygen concentration [7, 8]. High level of inflammation and infection and lower level of antioxidants in these infants lead to development of CLD. Tissue injury is also caused by oxidative stress following irradiation and hyperoxia. Both hypoxia and hyperoxia are contributors to increase in ROS. Abundant evidence has demonstrated that oxidative stress is a key player in ischemia due to oxygen reperfusion injury following hypoxia, leading to both cardiovascular diseases and strokes [9, 10]. Excessive free radicals are found in patients of chronic fatigue syndrome (CFS) [11]. Diabetes initiated due to hyperglycemia has a wide spectrum of disease manifestations as it leads to increased susceptibility of oxidative stress ultimately damaging various organs [12–16]. Endothelial cell dysfunction in diabetic patients is also associated with less production of nitric oxide (NO) important in enabling relaxed state of arterial vessels. Upon administration of antioxidants like SOD and catalase, endothelial cell function is improved indicating oxidative stress as an important secondary factor in diabetes [12].

## 3. Oxidative Stress in Neurodegeneration

One of the most metabolically active organs of the body is the brain, including the spinal cord comprising central nervous system (CNS), which, even at rest, utilizes an estimated 20% of the total oxygen uptake. During active state this percentage substantially increases and in order to carry out normal physiological actions, it requires an uninterrupted oxygen-rich blood supply. Any blockage or deprivation of this oxygen supply even for a few seconds can have severe and irreversible detrimental effect to the cells of brain (both neurons and glia). Consumption of oxygen leads to production of free radicals and the brain requirement for higher amount of oxygen leads to even higher number of reactive oxygen/nitrogen species. Though CNS has high requirement for oxygen but unexpectedly, is relatively deficient in the enzymes that metabolize a number of oxygen-based reactants to innocuous species [17]. On the contrary, CNS is highly enriched with polyunsaturated fatty acids which are readily oxidized by toxic oxygen derivatives [18]. Additional disadvantage is the presence of the blood-brain barrier, which is designed to protect the brain from toxins by limiting their diffusion into neurons and glia but also prevents/reduces the uptake of some antioxidants like vitamin E, into the brain. These features create an additive effect making the neurons and glia highly susceptible to destruction by free radical species. Progressive loss/damage (by these radicals) of structure and function of neurons caused is called as neurodegeneration. Being the power house of the cell, mitochondria are directly associated and susceptible to oxidative stress. This is not only due to its association with ROS but also due to the fact that mitochondria is not protected with histones and is inefficiently repaired leading to high mutation rates in mitochondrial DNA (mtDNA). There has been increasing evidence suggesting that mutations acquired during ageing by mtDNA contribute to physiological decline occurring with age and age-related neurodegeneration [7]. Therefore, oxidative stress is an important factor in neurodegenerative diseases, as the damage of the neurons could be due to either an increase in oxidative process or a decrease in anti-oxidant defenses or both. Although it is extremely difficult to distinguish whether mitochondrial-derived oxidative stress is the primary cause of toxicity or just reflect the consequence of neurodegeneration, recent evidences indicate that oxidative stress is the primary event [7].

Among others the three main neurodegenerative diseases are Alzheimer's disease (AD), Parkinson's disease (PD), and amyotrophic lateral sclerosis (ALS) which are not only sporadic, but also have rare familial forms. These diseases occur mainly as sporadic (90%) where as the familial cause is usually limited (10%). Sporadic forms occur due to a complicated and heterogeneous mixture of hereditary, environmental factors, and lifestyle stresses. In contrast Huntington's disease (HD), another neurodegenerative disorder is completely hereditary. HD is predominantly inherited and is strictly autosomal disorder. In the following sections role of oxidative stress in all the above-mentioned neurodegenerative disorders is briefly described. 

### 3.1. Oxidative Stress in Alzheimer's Disease

Clinically, Alzheimer's disease (AD) is characterized as a late-onset, age-dependent, progressive cognitive decline which results in irreversible loss of neurons especially in the cortex and hippocampus. The major pathological hallmarks of AD are the formation of extracellular senile plaques due to amyloid beta 42 peptide (A*β*42) aggregates and the intracellular neurofilament tangles (NFT) formed by hyperphosphorylated microtubule associated protein, tau (*τ*) [19]. In AD, oxidative stress has been associated as one of the earlier events. Due to oxidative stress, processing of APP or *τ* could be altered via activation of different signaling pathway.

Usually most of the AD cases are late onset and sporadic but there are 5–10% familial AD (FAD) occurring as an early-onset and in an autosomal-dominant manner. FAD can be caused due to mutations in three genes namely, amyloid precursor protein (APP), presenilin 1, and 2 (PS1, PS2). During normal physiological conditions proteolytic cleavage of APP is initiated by *α*-secretase followed by *γ*-secretase to yield nonamyloidogenic fragments [20]. Mutations in APP causes the altered proteolytic processing replacing *α*-secretase by *β*-secretase (BACE1), followed by *γ*-secretase to yield amyloidogenic A*β*42 which aggregates to form insoluble plaques. Various cell culture studies have shown A*β*42 causes toxicity and induces cell death via apoptosis [21]. 

In contrast to senile plaques, NFTs are so far not associated with mutations but are due to modulations of kinase and phosphatase activities. Normally *τ* functions to regulate microtubule (MT) assembly and transport. Where as in AD *τ* is hyperphosphorylated and dissociates from MT, resulting in destabilizing MTs and impairment of axonal transport. The phospho-*τ* aggregates form the paired helical filaments (PHFs) which further combine to form the insoluble NFTs [22]. Abnormal hyperphosphorylation of tau is a reflection of both an abnormal activation of kinases, as well as decreased phosphatase activity [23]. Among various kinases, cyclin-dependent kinase 5 (Cdk5) plays a major role in causing aberrant hyperphosphorylation (discussed later in this review). Pin 1 plays an important role in post phosphorylation of proline-directed Ser/Thr-residues involved in regulating protein function. In cell culture, inhibition of Pin1 reduces oxidative stress-induced apoptosis [24]. Conformational changes catalyzed by Pin1 affect both APP and tau processing. In animal studies, Pin1 knockout mice show increase in amyloidogenic APP processing thereby increasing the levels of A*β*42 and also exhibit tau hyperphosphorylation, neuronal degeneration, motor, and behavioral deficits [25, 26].

Hyperphosphorylated *τ* is one of the major causes of AD pathology as it not only fails in its normal function of stabilizing microtubules, but also exhibits a “gain of toxic function” due to its sequestering normal *τ*. Absence of normal *τ* results in the disruption of microtubules [27, 28]. Hyperphosphorylated *τ* accumulates to form the insoluble intraneuronal PHF-*τ* which could reflect inhibitory binding of oxidatively damaged protein to the proteasome. Under physiological conditions any aggregate which could be a potential toxic to the system is cleaned up by the proteasome. Inhibition of this cleanup process by the proteasome is sufficient to induce neuronal degeneration and death [29]. Also, various evidences suggest that at least in some part formation of the highly insoluble NFT is associated with oxidative stress [30, 31]. For the past two decades based on various mouse models and human clinical trials, much therapeutics are being employed to fight against AD starting from the use of antioxidants, neurotrophins, and statins to the use of hormone replacement and gene therapy [32]. However, the most effective drug treatment or regimen is yet to be determined as all the current therapies either have strong side effects or are not very effective.

### 3.2. Oxidative Stress in Amyotrophic Lateral Sclerosis

Amyotrophic lateral sclerosis (ALS) is clinically characterized by progressive weakness, atrophy, and spasticity of muscle tissue. Pathologically, ALS is an adult-onset neurodegenerative disease reflecting degeneration of upper and lower motor neurons in the cortex, brainstem, and spinal cord [33]. Like AD, ALS also occurs as both minor familial forms (fALS) and major (about 90%) sporadic forms (sALS). The major genetic defect accounting for about 3–20% of fALS is due to the mutation in the gene encoding ubiquitous enzyme Cu/Zn-superoxide dismutase (SOD-1). There have been more than 100 mutations identified in SOD-1 genes. Besides SOD-1 some of the other genes related to fALS include Alsin (ALS2), VAMP-associate protein B (VAPB), dynactin (DCTN1), TAR DNA-binding protein 43 (TDP-43), fused in sarcoma protein (FUS), and lipid phosphatase FIG4 (FIG4). Studies carried out on the postmortem tissue from sALS and fALS patients clearly show an accumulation of oxidative damage to proteins, lipids, and DNA indicating a direct role of oxidative stress in ALS [34–36]. Also, ALS is multifactorial pathogenetic disease occurring due to not only oxidative stress but also excitotoxicity, aggregate formation, inflammation, growth factor deficiency, and neurofilament disorganization, making ALS a complex disease especially for its amelioration [37].

In order to better understand the ALS pathogenesis, most of the recent studies have focused on mouse models that express the mutant human SOD1 forms. These animals experience age-dependent motor neuron degeneration with cellular and biochemical damage to nerve fibers and spinal cord tissue as well as increased protein and lipid oxidation [38]. Transgenic mice overexpressing the G93ASOD1 mutation has impaired mitochondrial energy metabolism in the brain and spinal cord at the onset of the disease, also there is a transient increase in the vacuolar mitochondrial degeneration preceding motor neurons death [39, 40]. All these evidences point towards involvement of the mitochondrial abnormalities triggering the onset of ALS. Recent reports have shown evidence of localization of SOD1 to mitochondria only in affected tissues and preferentially for mutSOD1 and therefore modulate mitochondrial functions [41]. MutSOD1 may interfere with the elements of the electron transport chain (ETC) to generate toxic ROS via aberrant superoxide chemistry and to promote oxidative damage to mitochondrial proteins and lipids [42, 43]. MutSOD1 may also disrupt mechanisms by which mitochondria buffer cytosolic calcium levels. 

With evidences from the mutSOD1 mouse model, the most accepted theory is that the different point mutations create a misfolding defect, leading to small amyloid-like aggregates that appear in late stages of the disease. These misfolded aggregated proteins could produce toxic effect towards neurons, similar to the neurotoxicity that arises in other amyloidoses [44, 45]. Misfolded aggregates are insoluble and are not cleared by proteasomal degradation, therefore they eventually impair and ultimately overwhelm the system [46, 47]. MutSOD1 aggregates are accumulated in the outer mitochondrial membrane and may block the protein import machines, TOM and TIM [41]. Also, mutSOD1 species bind and sequester mitochondrial Bcl-2 and cytosolic heat-shock proteins rendering them unavailable for antiapoptotic functions [48, 49]. 

Similar to mutSOD-1, more evidence propose alteration in proteins related to mRNA metabolism leading to protein misfolding as a salient feature of ALS pathogenesis [50, 51]. In various sALS and fALS cases, abnormal subcellular distribution and cytoplasmic aggregation of TAR DNA binding protein (TDP-43) is widely reported along with frontotemporal lobar degeneration [52, 53]. Under normal conditions, TDP-43 regulates different processes related to gene expression including transcription, splicing, and mRNA stability through RNA and DNA binding activities [50, 51, 54]. Proteomic analysis of the TDP-43 revealed associations with proteins related to RNA metabolism [55]. Also, TDP-43 interacts with components of stress granules and under oxidative stress TDP-43 redistributes itself to stress granules; however, the biological relevance of these observations is not clear yet [55, 56]. Most ALS linked mutations in TDP-43 are mapped to the C-terminal glycine-rich region, which is involved in protein–protein interactions between TDP-43 and other ribonuclear proteins [57]. In ALS and frontotemporal lobar degeneration derived tissue, C-terminal fragments of TDP-43 are specifically accumulated and have a high tendency to aggregate into intracellular inclusions [52]. Transgenic mice expressing human mutant TDP-43 develop a progressive and fatal neurodegenerative disease reminiscent of ALS, showing motor neuron loss, motor impairment, muscular atrophy, axonal degeneration, and mitochondrial dysfunction [53, 58]. Also, specific neuronal populations in the frontal cortex and the spinal cord of this mouse model show accumulation of ubiquitinated protein aggregates, suggesting that expression of TDP-43 mutants results in accumulation of misfolded proteins [53].

The above data suggests that both a toxic gain of function due to protein aggregation/mislocation together with a loss of normal biological function upon mutations contribute to disease pathogenesis. There have been mixed results of *in vivo* studies in the generation of ROS and oxidative damage, and trials of antioxidant therapies have been disappointing [37]. If protein misfolding is the major cause of SOD1-ALS or TDP-43 associated pathogenesis, then the drugs that could stabilize SOD1 or other proteins against misfolding would be a better therapeutic approach. Like in AD, it is unclear as to what extent potential therapeutic strategies based on the mouse models will translate to treatment of the human disease.

### 3.3. Oxidative Stress in Parkinson's Disease

Parkinson's disease (PD) is a common neurodegenerative movement disorder and is clinically characterized by progressive rigidity, bradykinesia, and tremor. Pathologically PD is characterized by loss of melanin-pigmented nigral neurons accompanied by depletion of dopamine in the striatum and the presence of Lewy bodies [59, 60]. Lewy bodies are detergent-insoluble and eosinophilic filamentous intraneuronal inclusions usually positive for ubiquitin and *α*-synuclein [61]. Although many evidences suggest that there is not much difference between the sporadic and the rare familial forms of PD, yet the exact molecular mechanism of the pathogenesis is still unclear. Like other neurodegenerative disorders, mitochondrial dysfunction, oxidative damage, environmental factors, and genetic predisposition may all be involved together in both sporadic as well as familial PD. 

Linkage analysis has led to the discovery of pathogenic mutations or polymorphisms in mtDNA in nine genes that may account for as many as 5–10% of the cases of familial PD. Out of these genes, two are autosomal dominant (coding for *α*-synuclein and dardarin) and three are autosomal recessive (including parkin) [62, 63]. Other genes associated with mutations in PD include ubiquitin carboxy-terminal hydrolase L1, *DJ-1*, phosphatase and tensin homologue- (PTEN-) induced kinase 1 (*PINK1*), leucine-rich-repeat kinase 2 (*LRRK2*), the nuclear receptor *NURR1*, *HTRA2, *and *τ* [7]. These mtDNA mutations associated with PD could be large-scale rearrangements, point mutations, or microdeletions [64–68]. The normal physiological function of *α*-synuclein is to maintain synapse and plasticity but mutations causing overexpression of *α*-synuclein is neurotoxic, inducing apoptosis [69, 70]. *In vitro* there is a strong connection between *α*-synuclein fibrillization and *τ* fibrillization and more evidence indicate frequent disease overlap between the classical tauopathies (e.g., NFT formation in AD) and synucleinopathies (e.g., Lewy body formation in PD) [71–73].

Postmortem tissues from PD patients have shown evidence that a defect in complex I of the mitochondrial electron-transport chain in substantia nigra, resulting in 30–40% decrease in the activity may be the central cause of sporadic PD [74]. The decreased activity could be due to underproduction of certain complex 1 subunits, complex 1 disassembly, or self-inflected oxidative damage [75–77]. More evidence of oxidative stress and PD comes from examination of substantia nigra region of human PD brain showing oxidative damage to DNA and protein along with immunocytochemical evidence for protein nitration and glycation [60, 78–83]. Also the most important lipid oxidation product 4-hydroxy-2-nonenal (HNE) was modified in PD brains [84, 85]. One of the other causes of sporadic PD include point mutations in *α*-synuclein leading to deposition of Lewy bodies by altering posttranslational modification of *α*-synuclein [86]. Impaired mitochondrial function, increased oxidative stress, and enhanced nigral pathology induced by MPP+, a metabolite of MPTP (1-methyl 4-phenyl-1, 2, 3, 6-tetrahydropyridine) inhibiting complex 1 are the characteristics of the transgenic mice overexpressing *α*-synuclein [87]. On the contrary, *α*-synuclein null mice are resistant to MPTP indicating an important role in mediating toxic effects of MPTP [88].

Ubiquitin, another important protein found in Lewy bodies of sporadic PD is associated with proteolytic stress as ubiquitin-proteasome process is impaired by products of oxidative damage like HNE [59, 89]. This also relates to other genetic defects associated with familial PD; E3 ubiquitin ligase called parkin. Mitochondrial impairment and increased oxidative stress is observed in parkin-null Drosophila and mouse strains [90, 91]. Similar to mutations in parkin, mutations in DJ-1 and PINK1 are also associated with autosomal recessive juvenile PD [92–95]. Both, DJ-1 and PINK1 protect against cell death. DJ-1 acts as a negative regulator of PTEN tumor-suppressor protein, increasing the cell survival and also DJ-1 null mice are hypersensitive to MPTP and oxidative stress [93]. Like DJ-1, PINK1 over expression prevents apoptosis under basal and staurospoine-induced conditions [96].

Besides mutations, overexposure to dopamine or metals like iron (Fe) and manganese (Mn) also plays an important role in the pathology of sporadic PD. In cell culture, dopamine is toxic to PC12 cell via oxidative stress, leading to apoptosis [97]. Dopamine oxidation leads to the formation of a known neurotoxin: 6-hydroxy-dopamine (6-OHDA) [98]. Two stimulants methamphetamine (METH) and 3, 4-methylenedioxymetham-phetamine (MDMA) when taken at very high dose also cause dopaminergic neurotoxicity [99]. In PD brains, a very high level of iron is found which increases with the severity of the disease [100]. High iron overload could be due to sequestration by eosinophilic protein aggregates and also iron has been implicated in the promotion of *α*-synuclein aggregation [101].

From the above-stated various factors causing PD, it is clear that PD not only results from a complex interplay among genetic and environmental factors, but also aspects of mitochondrial dysfunction and oxidative stress play a very important role. For a better therapeutic strategy for PD, all the causes and their interplay need to be taken into consideration for a drug, targeting PD.

### 3.4. Oxidative Stress in HD

As previously mentioned, Huntington's disease (HD) is genetically inherited in an autosomal manner. Clinically HD is characterized by psychiatric disturbances, progressive cognitive impairment, and choreiform movements. Pathologically HD is characterized by loss of long projection neurons, resulting in a progressive atrophy of the caudate nucleus, putamen, and globus pallidus [102].

The HD mutation is an expansion of CAG trinucleotide repeat within exon 1 of the huntingtin (*HTT*) gene, a cytoplasmic protein of unknown function [103]. Because the CAG triplet codes for glutamine extension, upon mutation, the protein presents a polyglutamine tract at the N-terminus, leading to a conformational change of the protein and ultimately resulting in abnormal protein-protein interaction. In humans, adult-onset HD occurs when there are more than 40 CAG repeats, compared to the normal number which is less than 36 repeats. In juvenile cases the expansion goes up to 70 repeats or more [104]. Mutant HTT confers a dominant “gain of function” to the protein, due to expanded polyglutamine segment, ultimately leading to neurodegeneration [105]. Various lines of evidence indicate that one of the major consequences of the gene expansion may be mitochondrial metabolic defect resulting in impaired energy metabolism [106]. Impaired mitochondrial energy possibly increases more production of free radicals which in turn leads to an increased oxidative damage.

Many recent evidences from HD patients indicate involvement of mitochondrial dysfunction in the pathogenesis. HD patient brains reveal increased production of lactate in the cerebral cortex and basal ganglia upon nuclear magnetic resonance imaging spectroscopy [107, 108]. Another study reported decreased complex I activity and no change in the activities of complexes II-III and IV in platelets [109]. Biochemical studies of brain tissue from human HD brain have shown multiple defects in the caudate: decreased complex II and complex II-III activity with no alteration of complex I or IV activities [110, 111]. Also ultrastructural abnormalities in mitochondria have been described in HD cortical tissue [112, 113]. Although oxidative stress does not have a very profound effect in HD compared to other major neurodegenerative diseases, HD patients exhibit low activity of catalase in skin fibroblast cultures [114]. In studies carried out on mutant HTT-knock-in mouse embryos, significant impairment in mitochondrial respiration and ATP production was observed [115].

In summary, mutant HTT causes abnormal protein interactions affecting normal mitochondrial function leading to oxidative stress and other downstream excitotoxic and inflammatory events, together resulting in neuronal death.

## 4. Deregulation of Cdk5 due to Oxidative Stress Leading to Neurodegeneration

Aberrant phosphorylation caused by the deregulated activity of cyclin dependent kinase 5 (Cdk5) has been closely associated with various neurodegenerative diseases like AD, ALS, HD, and PD. Cdk5, a proline-directed serine/threonine kinase, plays multiple roles in neuron development, neuronal survival, phosphorylation of cytoskeletal proteins and synaptic plasticity [116, 117]. The active form of Cdk5 is found primarily in the nervous system due to its activator proteins p35 or p39 specifically expressed in neuronal cells [118–120]. Activity of Cdk5 is tightly regulated and it plays an important role in CNS development by phosphorylating the specific serine or threonine site of numerous substrate proteins that are closely associated with the neuronal migration, synaptogenesis, and synaptic transmission as well as synaptic plasticity. However, under various stressed conditions like oxidative stress, mitochondrial dysfunctions, excitotoxicity, A*β* exposure, calcium dyshomeostasis, and inflammation lead to rise in the intracellular Ca^2+^. High Ca^2+^ concentration activates calpain which cleaves p35 to p25 forming a more stable yet hyperactive Cdk5/p25 complex [119, 121–128]. Various cytoskeletal proteins are aberrantly hyperphosphorylated by this complex eventually leading to neuronal death/neurodegeneration [122, 123, 129–131]. 

### 4.1. Role of Cdk5 in AD

Cdk5 is activated by oxidative stress in AD, resulting in hyperactive and aberrant Cdk5/p25 activity causing hyperphosphorylation of *τ*, neurofilament (NF) and other cytoskeletal proteins [123, 132, 133]. Oxidative stress and mitochondrial dysfunction are one of the earliest events in AD pathology preceding appearance of NFT [134]. Accumulation of A*β* in cortical neurons induces cleavage of p35 to p25 resulting in activation of kinases and inhibition of phosphatases proceeding NFT formation [21, 123]. Upon neuronal insult with either A*β* or glutamate, primary cortical neurons have shown enhanced Cdk5 activity. After treatment with inhibitors like roscovitine or Cdk5 inhibitory peptide (CIP), cells displayed reduction in hyperactive Cdk5 [21, 135, 136]. Also, cell culture results have shown that A*β* and glutamate toxicity cause Cdk5 to promote mitochondrial damage and induce p38 activation by increasing ROS [137, 138]. In AD brains significant increase in p25 levels and activity of Cdk5 and p38 is observed [139, 140]. Furthermore, mitochondrial depolarization results in more ROS formation and Ca^2+^ release, both of which eventually activate Cdk5 [122, 127]. Therefore, hyperactivity of Cdk5 is involved in promoting cell death via a feedback loop mechanism by being an upstream regulator as well as a downstream effector of mitochondrial dysfunction [137]. More evidences from cell culture studies have shown various substrates of Cdk5 leading to more ROS production. Two such substrates are peroxiredoxin-I (Prx-I) and peroxiredoxin-II (Prx-II) belonging to the Prx family of peroxidases that under physiological conditions efficiently scavenge ROS [141–144]. Cdk5-mediated phosphorylation of Prx-I and Prx-II reduces their enzymatic activities resulting in ROS accumulation within the cells [137].

Silencing Cdk5 via RNA interference (RNAi) using lentiviral or adenoassociated viral vectors in the brains of transgenic AD mouse models show reduction in phosphorylation of tau and decreased number of NFTs in the hippocampus [145]. Besides being involved in hyperphosphorylation of tau and formation of NFTs, Cdk5/p25 has also been involved in phosphorylation of APP in its cytoplasmic domain at Thr668 [146]. Increased APP Thr668 phosphorylation has been observed in p25 transgenic mice compared to normal p35/Cdk5 activity [147]. These studies taken together strongly suggest that Cdk5 activation may be an early event in AD and therefore, could be used as a potential therapeutic target for AD.

### 4.2. Role of Cdk5 in ALS

Aberrant Cdk5 hyperactivity due to oxidative stress is also linked to motor neuron degeneration and ALS by compromising the NF dynamics [129]. In cultured neurons, when exposed to oxidative stress via treatment with hydrogen peroxide, Cdk5 phosphorylated the high molecular weight NF (NF-H). This inhibited NF axonal transport and induced perikaryal accumulation of NF phosphoepitopes normally confined to axons [148–150]. Upon inhibition of Cdk5 activity with either roscovitine or CIP, these effects were prevented indicating role of Cdk5 in ALS pathology [127]. Mutations in SOD1 are associated with ALS and promote increased oxidative stress and increased production of ROS [151, 152]. Transgenic mice expressing mutant human SOD1 display increased ratio of p25/p35 in addition to abnormal localization and hyperactivation of Cdk5 [153]. Also, these mice have perturbed axonal transport and display aberrant NF accumulation within perikarya [154]. All these evidence suggests a major role of oxidative stress-induced Cdk5 hyperactivity leading to perikaryal NF phosphorylation inhibiting NF axonal transport causing motor neuron degeneration.

### 4.3. Role of Cdk5 in PD

Evidence of Cdk5 hyperactivity associated with PD comes from the studies involving gyrus cinguli (brain region above corpus callosum) of PD patients showing higher p25/p35 ratio due to calpain activation compared to age-matched controls [155]. As previously stated parkin phosphorylation and ubiquitination may modulate the formation of the Lewy bodies, relevant to the disease. Transgenic mouse model studies have indicated Cdk5 as a new regulator of the parkin ubiquitin-ligase activity since serine 131 has been identified as the major Cdk5 phosphorylation site in parkin. The Cdk5 phosphorylation-deficient S131A parkin mutant displays increased autoubiquitination and is more prone to aggregation upon proteasome inhibition. Furthermore, this mouse increases the formation of synphilin-1/*α*-synuclein inclusions indicating that phosphorylation of parkin by Cdk5 decreases its E3 ubiquitin-ligase activity and regulates the formation of cytosolic inclusions relevant to PD [156]. Phosphorylation by Cdk5 may contribute to the accumulation of toxic parkin substrates and decrease the ability of dopaminergic cells to cope with toxic insults in PD.

### 4.4. Role of Cdk5 in HD

Contrary to the role of Cdk5 in above-mentioned neurodegenerative diseases like AD, ALS, and PD, Cdk5 shows a neuroprotective role in HD. As mentioned earlier cause of HD is the expansion in polyQ stretch in the huntingtin (htt) protein. More than 40 polyQ tracts leads to htt protein misfolding, making it toxic and resulting in formation of aggregates and cause disease. Inhibiting polyQ aggregation alleviates the symptoms of HD patients, as reported in *Drosophila* and mouse models of HD [157, 158]. Short fragments of htt with expanded polyQ repeats show greater toxicity and aggregation compared with full-length mutant constructs and are sufficient to cause cell death in cell culture or disease in animal models [159, 160]. Thus, mutant htt cleavage either by caspases, calpains, or other proteases resulting in toxic fragment production may be an important rate-limiting step in HD pathogenesis [160–162]. Various evidences have shown that htt not only interacts and colocalizes with Cdk5 in cellular membrane fractions but is also phosphorylated at Serine 434 leading to reduced caspase-mediated htt N-terminal cleavage at residue 513, resulting in decreased aggregation [163]. Also, Cdk5 phosphorylates at Serine1181 and Serine1201 to prevent the gain of toxic activity [164]. These results also suggest the ability of Cdk5 phosphorylation to protect against htt cleavage, aggregation and toxicity, which are compromised in cells expressing toxic fragments of htt [163]. Another study carried out on cell lines and rat or mouse cortical neuron cultures shows the kinase activity of Cdk5/p35 suppressing inclusion formation of polyQ proteins by disrupting microtubules (MTs). Cdk5-dependent regulation of MT organization is involved in the development of aggregate formation and subsequent pathogenesis of polyQ diseases [165]. This Cdk5 inhibition of htt aggregates offers a novel mechanism and hence could be used as a potential therapeutic target for HD. 

**Figure 1:**
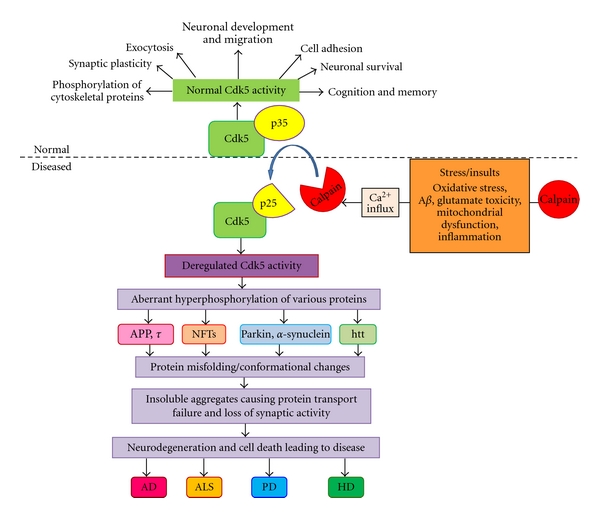
Summary of role of Cdk5 in physiology and pathology. Cdk5 is a proline-directed serine/threonine kinase and gets activated by its neuron specific promoter p35. Under normal physiological (normal) conditions, Cdk5/p35 is involved with various roles in neuronal development, cognition and memory, cell adhesion, phosphorylation of cytoskeletal proteins, and synaptic plasticity. When the neurons are stressed due to various insults like oxidative stress, inflammation, mitochondrial dysfunction, or toxicity due to A*β* or glutamate, there is an increase in Ca^2+^ leading to activation of calpain, a protease. Calpain cleaves p35 to p25 deregulating Cdk5 activity as p25 forms a hyperactive and more stable complex with Cdk5. Cdk5/p25 activity causes aberrant phosphorylation of various proteins leading to conformational changes inducing gain of toxic function. Misfolded proteins lead to self-aggregation thereby overwhelming the system by blocking transport and disrupting synaptic activity. This ultimately leads to degeneration of neurons and finally be the cause of various neurodegenerative diseases like AD, ALS, PD, and HD.

Figure 1 describes in brief the role of Cdk5 in physiology as well as in pathology. Under healthy physiological conditions, Cdk5 plays a very important role in various neuronal functions. However, due to various stress and insults, deregulated Cdk5 activity leads to pathological condition.

## 5. Conclusions

Recent evidences have greatly increased our knowledge about the AD, ALS, PD, and HD, major neurodegenerative diseases assessed above. From various studies, it has become evident that all the neurodegenerative diseases are to some extent multifactorial, and oxidative stress is inevitably intertwined with the disease mechanisms. Besides biological factors like inflammation, excitotoxicity, and to a certain extent role of genes involved in sporadic cases, environmental contributions like diet and lifestyle are also important contributing factors for the occurrence of these diseases.

These disorders have many common factors. One such factor is the deregulation of Cdk5 as depicted in Figure 1. Cdk5 plays a very important role in these neurodegenerative disorders. In a normal physiological condition, Cdk5 along with its neuron specific activator p35 is involved in various neuronal processes necessary for normal function. Under neuronal stress and insults which could be due to many factors like oxidative stress and release of ROS, A*β* or glutamate toxicity, inflammation, and mitochondrial dysfunction, there is intracellular increase in Ca^2+^ concentration. This leads to the activation of proteases-like Calpain which cleaves p35 into p25. Association of p25 with Cdk5 leads to the diseased condition as Cdk5/p25 activity is deregulated causing aberrant hyperphosphorylation of various proteins like neurofilament, *τ*, parkin, *α*-synuclein, and Huntingtin. Hyperphosphorylation of these proteins leads to misfolding and aggregation causing conformational changes. Misfolded proteins self-aggregate disrupting transport and normal synaptic activity ultimately leading to neuronal degeneration and pathology.

Since oxidative stress is involved in neurodegeneration, selecting antioxidants, metal chelators, or other compounds boosting endogenous enzymatic and nonenzymatic defense mechanism seems to be an obvious choice as a treatment to these disorders. However, using antioxidants like Vitamin E (tocopherol) and others as a therapeutic target comes with a caveat as most antioxidants have metal-reducing capacity. Therefore, devising a successful regimen of antioxidants to retard the progression of these diseases remains a complicated goal. Besides using anti-oxidants as therapeutic targets, Cdk5 with all its involvement in the above mentioned neurodegenerative diseases seems to be potential and ideal candidate to be used as therapeutic target for neurodegenerative diseases.
